# Comparison of Root Coverage by the Subepithelial Connective Tissue Graft With and Without Root Biomodification: A Comprehensive Clinical Study

**DOI:** 10.7759/cureus.44758

**Published:** 2023-09-06

**Authors:** Khushboo Kumari, Barsha Nath, Amrit Kumar, Amarpal Kour Chhabada, Rosy Kumari, Gyan Prakash

**Affiliations:** 1 Department of Oral Pathology, Buddha Institute of Dental Sciences and Hospital, Patna, IND; 2 Department of Periodontology and Implantology, Buddha Institute of Dental Sciences and Hospital, Patna, IND; 3 Department of Dentistry, Gandhi Dental College, Utkal University, Bhubaneswar, IND

**Keywords:** subepithelial connective tissue graft, root coverage, root conditioning, root biomodification, gingival recession

## Abstract

Background: Being an autologous graft, a subepithelial connective tissue (SECT) graft shows more predictable root coverage better than other techniques. Hence, it is most likely to be widely used for recession treatment. During root planing, a smear layer forms on the root surface that cannot be removed by water or saline rinsing. To remove this smear layer, root biomodification agents are widely used. The present study was conducted to assess the efficacy of an SECT graft for root coverage with and without root biomodification.

Methods: This study included 20 patients with no gender predilection, with an age range of 24-36 years and a mean age of 27.6 ± 4.24 years. The chosen range facilitated the acquisition of data in a relatively homogeneous population, minimizing the confounding effects of factors such as aging-related tissue changes or early-onset periodontal issues. All 40 sites were treated with the SECT and coronally advanced flap. Root conditioning in controls was done with distilled saline and tested using 24% ethylenediaminetetraacetic acid (EDTA) gel (Maquira; STM Meditech, Kerala). At baseline and at one, three, and six months postoperatively, pocket depth (PD) and clinical attachment levels (CALs) were assessed at four sites using a UNC-15 probe, and from the gingival margin to the cementoenamel junction (CEJ), the vertical recession was assessed.

Results: For the buccal surface, CALs were reduced significantly (p < 0.001). Following root conditioning with 24% EDTA, no difference was seen in the CAL in the control and test group either buccally or interproximally with a p-value of greater than 0.05. For PD, following a SECT graft or root conditioning, no significant change was observed in the buccal or interproximal region (p > 0.05). The vertical recession was significantly reduced with a p-value of less than 0.001 and depth coverage of 97.5%. The difference between the two groups was statistically non-significant (p > 0.05). The root surface coverage decreased significantly from 16.6 ± 2.8 to 0.45 ± 0.4 from baseline to six months, which was statistically significant (p < 0.001). This intergroup difference was non-statistical (p > 0.05).

Conclusion: The present study concludes that the use of an SECT graft in root coverage can significantly improve the CAL, root surface area, and vertical recession both with and without root biomodification. We conclude that there is a significant decrease in the probing depth following SECT grafting and with root biomodification.

## Introduction

Gingival recession is a common periodontal disease characterised by the migration of the gingival margin apical to the cementoenamel junction (CEJ). It is caused by a variety of factors, such as tooth loss, orthodontic treatment, occlusal disharmony, plaque accumulation, and improper tooth brushing. Recession can lead to various difficulties, including tooth loss, difficult oral hygiene, dentinal hypersensitivity, and aesthetic concerns, when anterior teeth are affected, necessitating its treatment. Over the years, recessions have been treated with different techniques and methods. However, subepithelial connective tissue (SECT) graft, along with a few other techniques, is one of the most common ways to cover up a recession. It is also considered the “gold standard” [1].

Being an autologous graft, an SECT graft shows more predictable root coverage along with aesthetic harmony compared to other techniques. Hence, it is the gold standard for recession treatment, which was first used by Langer and Langer [2]. The most common parameter used to assess the success of recession coverage is gingival height, an increase in which is considered a successful treatment following augmentation. The high success rate of an SECT graft can be attributed to the dual blood supply from the graft and flap. Previous studies have revealed that an SECT graft has a high success rate in achieving a recession coverage of 2-6 mm. Before root coverage, the denuded root surfaces are planned using either ultrasonic or manual instruments to remove diseased cementum and calculus to achieve a clean root surface that is biologically acceptable to allow attachment formation and promote cell growth. However, during root planing, a smear layer forms on the root surface, which cannot be removed by water or saline rinsing [2-4]. A hypermineralised surface layer can also be seen associated with the root surfaces exposed by the recession. For the removal of this smear layer, root biomodification agents are widely used, ethylenediaminetetraacetic acid (EDTA), citric acid, and tetracycline being the common ones. In vivo studies have shown that being a chelating agent, EDTA acts at a neutral pH and leads to early cell colonisation and collagen fibre exposure. Neutral pH etching can also help preserve the vitality of the adjacent tooth [4].

A gingival recession leads to various aesthetic and functional concerns. High incidences of hypersensitivity, clinical attachment loss, and root caries are also associated with marginal gingival recession cases. The treatment of gingival recession aims at providing an adequately attached gingiva zone, preventing root caries, promoting plaque retention, and improving aesthetics [1,4]. Since the time of the mid-20th century, various techniques have been tested and developed to cover single and multiple gingival recessions. With the introduction of SECT grafts in mucogingival surgical procedures, this predictability has significantly improved with root coverage procedures. Different root coverage procedures have become popular because they are easy to predict and have a high success rate [2,3].

In previous literature studies, smear layer formation was assessed after different root planing techniques by evaluating the effect of EDTA on different clinical parameters. Following the scanning electron microscopy examination, 24% EDTA was found to be effective in removing the smear layer formed after root planing [5]. The use of EDTA has also been suggested as an adjuvant with mechanical root planing and irrigation with ultrasonic scalers. Other than collagen exposure and smearing layer removal, studies have found no additional benefit of EDTA root conditioning. With different available concentrations, only 15% and 24% EDTA were able to completely remove the smearing layer [6]. The goal of this study was to find out how well an SECT graft works for root coverage with and without biomodification of the roots.

## Materials and methods

The present prospective clinical study was conducted to assess the efficacy of an SECT graft for root coverage with and without root biomodification. The study subjects were from the Department of Periodontology of the Buddha Institute of Dental Sciences and Hospital (ethical approval no. BIDSHP/2021/109). The study included 20 subjects of both genders within the age range of 24-36 years and the mean age of 27.6 ± 4.24 years. Those with a plaque index of less than 0.5 (good hygiene), Miller class I or II recession, non-smokers, systemically healthy patients, and those who had opted for treatment with an SECT graft were included in the study. Those who were unwilling to provide consent or participate in the study, were tobacco users, and had carious teeth, restored teeth, systemic medication, and occlusal trauma were excluded. After explaining the detailed study design, informed consent was obtained from all the study subjects. All 40 sites were treated in the 20 subjects by a single periodontist expert in the field.

**Figure 1:**
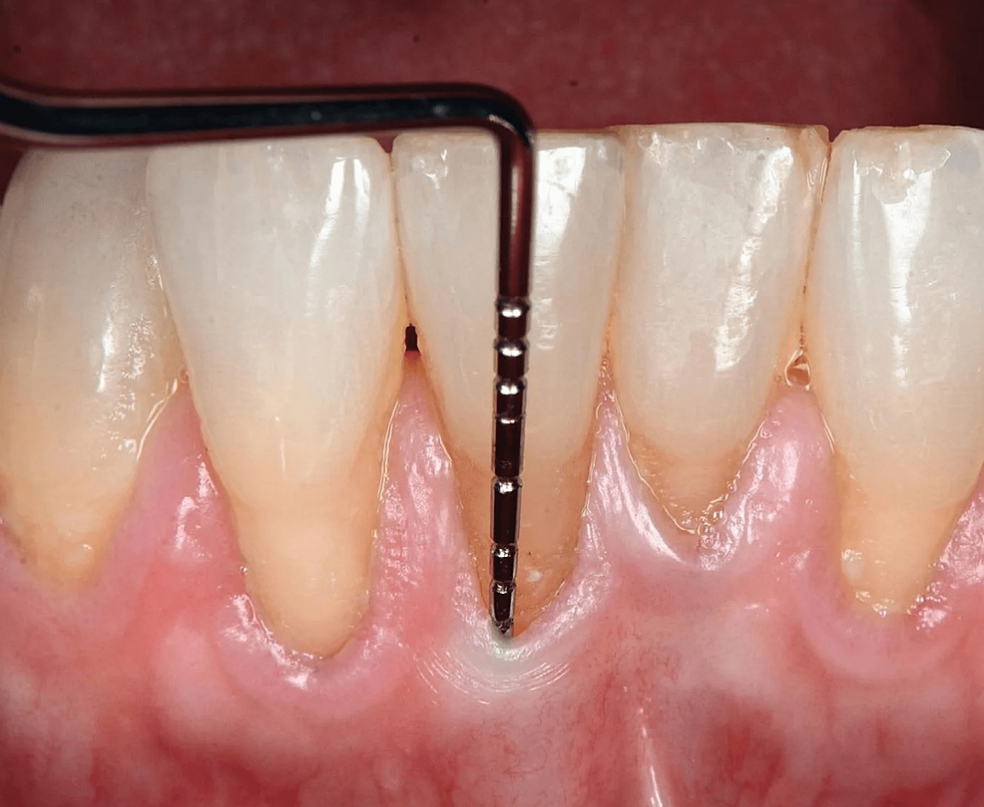
Preoperative measurements of the probing depth and clinical attachment loss

After the final inclusion, the recession sites were randomly divided into two groups (n = 20), namely, controls and tests; control sites were treated with distilled saline and test sites with 24% EDTA gel (Maquira; STM Meditech, Kerala). Patients were assigned to two groups and the operator was blinded to the procedure to be done. After the scaling and root planing was done, patients were asked to follow routine oral hygiene instructions and then were recalled after four weeks for further surgery. At baseline and six months, postoperatively, pocket depth (PD) and clinical attachment levels (CALs) were assessed at four sites using a UNC-15 probe, and from the gingival margin to CEJ, the vertical recession was assessed. The root surface coverage (RSC) percentage along with the surface area of the recession was assessed at baseline and six months, using models (Figure 1). The following formula was used: percentage root surface coverage = total root surface length/covered root surface length × 100%, where covered root surface length refers to the length of the root surface that is covered or treated and total root surface length refers to the total length of the root surface being considered.

**Figure 2:**
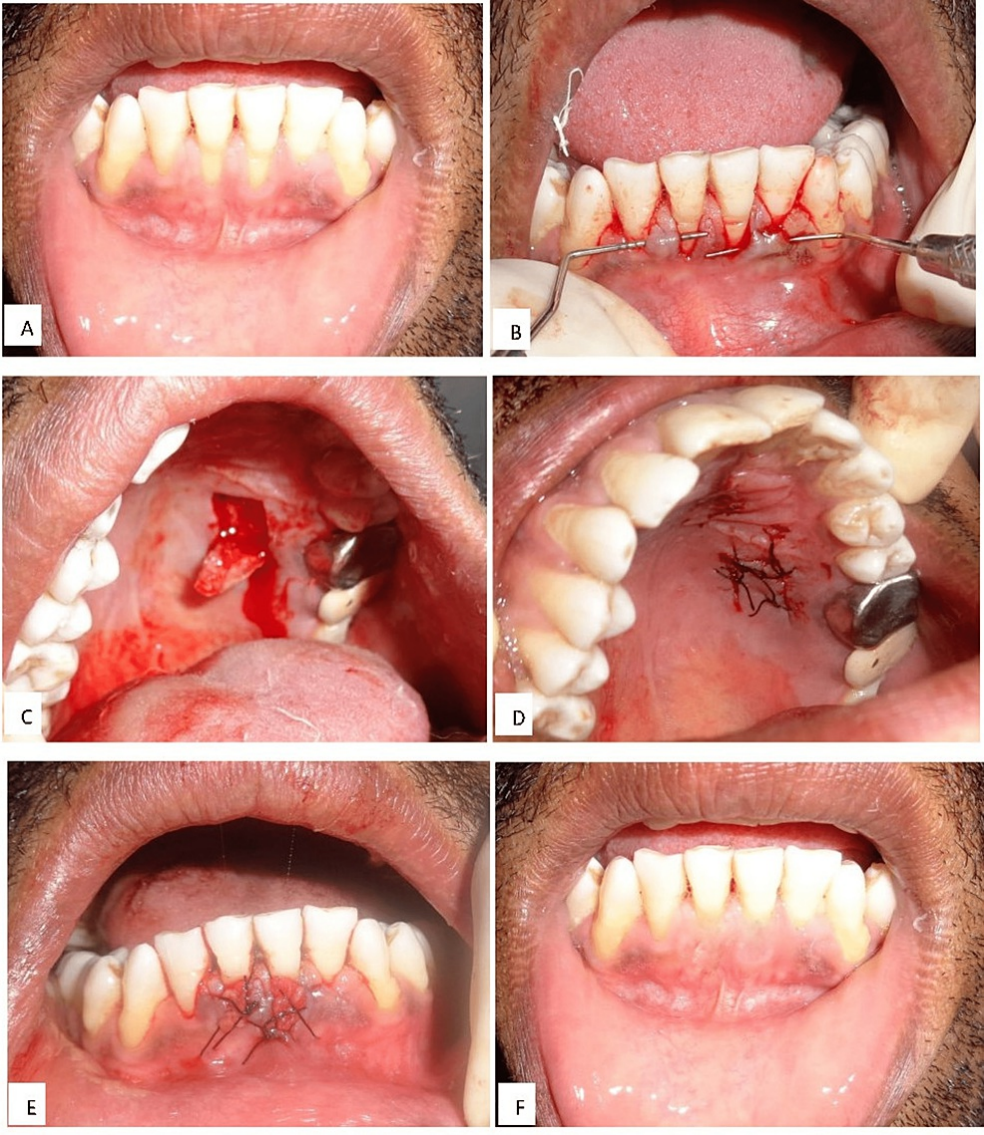
Surgical technique at various stages (A) Preoperative view, (B) root biomodification, (C) graft from the donor site, (D) suturing at the donor site, (E) grafting at the surgical site, (F) at postoperative three months

At all 40 sites, Langer and Langer's technique was used to cover gingival recession using an SECT graft and coronally advanced flap under local anesthesia. Before flap reflection, root surfaces were conditioned for five minutes after curetting them. In controls, roots were conditioned with distilled saline and tested using 24% EDTA gel at pH 7.4. For the donor site, under local anesthesia, the horizontal incision was made 5-6 mm from the gingival margin on the palatal premolar area, which was connected with two vertical incisions to harvest an SECT graft of 1.5 mm. The harvested graft was placed on the recession site and was sutured. Following surgery, postoperative instructions were provided along with required medications. The subjects were recalled after one week for suture removal and were further assessed at one, three, and six months (Figure 2).

**Figure 3:**
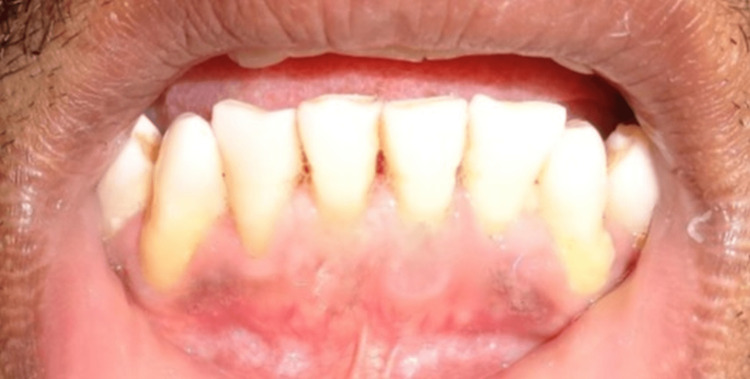
Postoperative healing at six months

Postoperative healing after six months was also uneventful (Figure 3).

The collected data were assessed for result fabrication and were expressed as means, percentages, and numbers. The level of significance was kept at a p-value of less than 0.05.

## Results

The study included 20 subjects from both genders, with 40 sites; the age range was 24-36 years, with a mean age of 27.6 ± 4.24 years. At the mid-buccal and interproximal sites, CALs were assessed using CEJ as a reference. Following an SECT graft, no difference was seen in interproximal CALs, whereas, for the buccal surface, CALs were reduced significantly from 5.7 ± 0.7 to 1.7 ± 0.7 mm postoperatively (p < 0.001) (Table 1).

**Table 1:** Preoperative and postoperative (six months) clinical parameters following an SECT graft in the study subjects CAL, clinical attachment level; SECT, subepithelial connective tissue; RSC, root surface coverage

Parameters	Subgroup	Preoperative (mm) (n = 40)	Postoperative (mm) (n = 40)	p-value
CAL	Buccal	5.7 ± 0.7	1.7 ± 0.7	<0.001
	Interproximal	2.2 ± 0.6	2.0 ± 0.3	NS
Probing depth	Buccal	1.6 ± 0.6	1.5 ± 0.6	NS
	Interproximal	2.1 ± 0.0	2.1 ± 0.0	NS
Vertical recession depth		4.1 ± 0.6	0.1 ± 0.2	<0.001
Percentage vertical recession depth		-	97.5%	-
Root surface coverage		16.6 ± 2.8	0.45 ± 0.4	<0.001
Percentage RSC		-	97.2%	-

Following root conditioning with 24% EDTA gel, no difference was seen in CALs in the control and test groups, neither buccally nor interproximally, with a p-value of greater than 0.05 (Table 2).

**Table 2:** Preoperative and postoperative (six months) clinical parameters with and without root conditioning in the study subjects CAL, clinical attachment level; EDTA, ethylenediaminetetraacetic acid; RSC, root surface coverage

Parameters	Subgroup	EDTA (mm) (n = 20)	Controls (mm) (n = 40)
CAL (preoperative)	Buccal	6.1 ± 0.6	5.7 ± 0.4
	Interproximal	2.4 ± 0.4	2.4 ± 0.4
CAL (postoperative)	Buccal	1.8 ± 0.8	1.5 ± 0.6
	Interproximal	2.1 ± 0.5	2.0 ± 0.0
Probing depth (preoperative)	Buccal	1.8 ± 0.4	1.7 ± 0.4
	Interproximal	2.1 ± 0.0	2.1 ± 0.0
Probing depth (postoperative)	Buccal	1.7 ± 0.4	1.7 ± 0.4
	Interproximal	2.1 ± 0.0	2.1 ± 0.0
Vertical recession depth (preoperative)		4.4 ± 0.4	4.1 ± 0.4
Vertical recession depth (postoperative)		0.2 ± 0.2	0.2 ± 0.2
RSC preoperative		17.4 ± 1.8	15.8 ± 3.1
RSC postoperative		0.4 ± 0.4	0.3 ± 0.2
Percentage vertical recession depth		97.6	97.4
Percentage RSC		97.2	97.6

For PD, following the SECT graft, the probing depth did not change significantly from baseline to six months in the buccal or interproximal region (p > 0.05). Following root biomodification, in the buccal area, PD reduced from 1.8 ± 0.4 to 1.7 ± 0.4, whereas in the interproximal area, it remained at 2.1 ± 0.0. PD did not change in the control group either buccally or interproximally. The difference was statistically non-significant (p > 0.05). The probing depth was reduced in the root surfaces following root biomodification, showing the efficacy of root biomodification or conditioning in the cases of gingival recession undergoing root coverage. On assessing the vertical recession in the study subjects, the vertical recession depth was seen to have decreased significantly from 4.1 ± 0.6 to 0.1 ± 0.2 mm postoperatively following the SECT graft with a p-value of less than 0.001 with a percentage vertical recession depth coverage of 97.5% (Table 1). In the test group, after 24% EDTA gel, the vertical recession depth decreased significantly from 4.4 ± 0.4 to 0.2 ± 0.2 mm and in the control group from 4.1 ± 0.4 to 0.2 ± 0.2 mm. The difference between the two groups was statistically non-significant (p > 0.05). The percentage vertical recession depth was 97.6% and 97.4%, respectively, for the test and control groups, which had statistically non-significant differences (p > 0.05) (Table 2). The study results remained consistent with the view that the SECT graft is a gold standard procedure for root coverage procedures. These results also showed that root biomodification in addition to root coverage yields results that are clinically superior to those achieved through the root coverage procedures alone.

Concerning the RSC following the SECT graft, it decreased significantly from 16.6 ± 2.8 to 0.45 ± 0.4 from baseline to six months, which was statistically significant (p < 0.001). The percentage RSC was 97.2%, as shown in Table 1. The preoperative RSC in the test group was 17.4 ± 1.8, which significantly reduced postoperatively to 0.4 ± 0.4. A similar statistical difference was seen in the control group: from 15.8 ± 3.1 to 0.3 ± 0.2 postoperatively at six months. This intergroup difference was statistically non-significant (p > 0.05). The percentage root coverage in the test and control groups was 97.2% and 97.6%, respectively, which was statistically non-significant between the two groups (p > 0.05) as depicted in Table 2. It was seen that RSC yielded good results when SECT grafts were used for root coverage procedures, with significant results reported with root biomodification compared to the controls, showing superior results of root biomodification with gold standard SECT grafts.

## Discussion

The present study was conducted to assess the efficacy of an SECT graft for root coverage with and without root biomodification. For the exposed root surfaces with a recession, the root coverage procedures and mucogingival surgical procedures were aimed at fulfilling the functional and aesthetic concerns associated with the gingival recession. Root conditioning or root biomodification done before root coverage procedures can be either chemical, mechanical, or a combination of both. Chemical methods of root biomodification depend mostly on acid agents, which are thought to work by making the root surfaces less mineralized when the gingiva recede.

In this study, 40 sites in 20 subjects with a mean age of 27.6 ± 4.24 years and an age range of 24-36 years were treated with an SECT graft either with 24% EDTA gel root biomodification or after saline application. The study results showed no difference postoperatively in the CAL interproximally. However, buccal CALs were significantly reduced postoperatively (p < 0.001). After the application of 24% EDTA for root biomodification, no difference was observed in CALs between the test and control groups in the interproximal and buccal areas (p > 0.05). PD did not reduce significantly at six months of assessment in both interproximal and buccal regions (p > 0.05). After 24% EDTA, PD in the buccal area decreased from 1.8 ± 0.4 to 1.7 ± 0.4. No such change was seen in the interproximal area or control group (p > 0.05). These results were consistent with the studies of Alves et al. and Alkan and Parlar, where the authors reported significant CAL reduction following an SECT graft and no difference in the PD or CAL following root biomodification [7,8]. These findings suggest that root biomodification using EDTA as a root biomodification agent along with the gold standard mucogingival recession coverage procedure yields clinically promising results concerning the coverage of the gingival recession.

Our study results showed a significant reduction in the vertical recession depth postoperatively after the SECT graft (p < 0.001) and a 97.5% vertical recession depth coverage. After root biomodification, the vertical recession depth decreased significantly in the test group from 4.4 ± 0.4 to 0.2 ± 0.2 mm and in the control group from 4.1 ± 0.4 to 0.2 ± 0.2 mm, with a non-significant intergroup difference (p > 0.05). The percentage vertical recession depth was 97.6% and 97.4% for the test and control groups, respectively (p > 0.05). These results are in agreement with the findings of Henriques et al. and Gruber et al. who reported a significant vertical recession reduction following an SECT graft and non-significant reduction following root biomodification [9,10]. These results show excellent predictability with the SECT grafts, with the added advantages of use in both single and multiple recession cases, good aesthetics, excellent color match, and blood supply to the graft, though with disadvantages of being time-consuming and technically challenging.

The RSC decreased significantly from 16.6 ± 2.8 to 0.45 ± 0.4 from baseline to six months with a p-value of 0.001 and a percentage root coverage of 97.2%. The postoperative RSC decreased significantly from 17.4 ± 1.8 to 0.4 ± 0.4 in the test group and from 15.8 ± 3.1 to 0.3 ± 0.2 in the control group (p < 0.001). However, the intergroup difference was non-significant (p > 0.05). These findings corresponded with the results of Shirakata et al. and Cairo et al., where the authors reported significant root coverage following an SECT graft and non-significant differences in the RSC with or without root biomodification [11,12]. The use of SECT grafts along with the gingival recession coverage showed clinically acceptable results in the root coverage for teeth, and these results were statistically significant. Root biomodification adds to the clinical success of the SECT graft, which is considered the gold standard in root coverage procedures [13-15].

One limitation of our study is that when the amount of time spent observing the study population increases, it leads to a longer term follow-up study. This is done in the event of more discrepancies developing between various treatment modalities. In order to evaluate our findings with regard to the recurrence rate, additional randomized clinical trials are required.

## Conclusions

The use of SECT grafts in root coverage yielded favourable results, which significantly improved the CAL, RSC, and vertical recession, regardless of whether or not root biomodification was performed. The RSC produced positive results when SECTs were used for the root coverage procedures, demonstrating superior root biomodification with gold standard SECT grafts. Following the SECT grafting, there was no discernible change in the PD. Regarding the coverage of the recession, root biomodification does not provide any additional benefit. The recent controversies underscore the need for robust and standardized research methodologies to elucidate the true impact of root biomodification on recession coverage outcomes.
